# Genetic liability to critically ill COVID-19 increased risk of HER2-positive breast cancer through the immune pathway: A Mendelian randomization study

**DOI:** 10.1097/MD.0000000000042372

**Published:** 2025-05-09

**Authors:** Jianfeng Chen, Bingyan Xin, Wenwen Tan, Zhilian Chen, Lan Zhang, Xiaochuan Zhu

**Affiliations:** a Department of Toxicology II, Hunan Prevention and Treatment Institute for Occupational Diseases (Affiliated Prevention and Treatment Institute for Occupational Diseases of University of South China), Changsha, China; b Department of Occupational Lung Diseases II, Hunan Institute of Occupational Disease Prevention, Changsha, China.

**Keywords:** breast cancer, COVID-19, immune cell, immune pathway, Mendelian randomization, SARS-CoV-2

## Abstract

The clinical management of patients with both coronavirus disease 2019 (COVID-19) and breast cancer remains a complex and unresolved issue that despite extensive discussion has not reached a consensus. In contemporary literature, the association between COVID-19 and HER2-positive breast cancer has received minimal attention. Genetic instruments for severe acute respiratory syndrome coronavirus 2 infection (N = 2,597,856), hospitalized COVID-19 (N = 2,095,324), and critically ill COVID-19 (N = 1,086,211) were obtained from the COVID-19 Host Genetics Initiative genome-wide association study (GWAS) meta-analysis. A total of 103,530 HER2-positive breast cancer cases from GWAS were enrolled in our study. The summary GWAS statistics of 731 immune cells (N = 3757) were obtained from the MRCEU open database. Causal associations were evaluated by applying Mendelian randomization (MR) including inverse variance weighting, MR-Egger regression, and weighted-median analysis. Sensitivity analyses were employed, including Cochran *Q* test, MR-Egger intercept test, MR Pleiotropy Residual Sum and Outlier, and leave-one-out analysis, to examine the robustness of these findings. Genetic liability to critically ill COVID-19 was significantly causally associated with the increased risk of HER2-positive breast cancer (odds ratios = 1.086, 95% confidence intervals = 1.015–1.162, *P* = .016). No causal associations between severe acute respiratory syndrome coronavirus 2 infection or hospitalized COVID-19 and HER2-positive breast cancer were observed. Additionally, genetic liabilities to critically ill COVID-19 were significantly positively associated with 9 immune cells. IgD- CD27- AC and CD27 on IgD+ CD38- unsw mem (memory B cells) were identified as potential mediators of the causal relationship between critically ill COVID-19 and HER2-positive breast cancer. Genetic liability to critically ill COVID-19 is associated with an increased risk of HER2-positive breast cancer possibly through the immune pathway. Future studies are essential to elucidate the mechanisms underlying this causal relationship, aiming to develop therapeutic strategies to mitigate the immune cell-mediated impact on HER2-positive breast cancer risk.

## 1. Introduction

Coronavirus disease 2019 (COVID-19), caused by severe acute respiratory syndrome coronavirus 2 (SARS-CoV-2), has rapidly spread globally since its emergence in 2019, leading to over 776 million confirmed cases and approximately 7.1 million deaths as of July 2024 (Source: World Health Organization).^[1]^ Post-COVID-19 infection, most individuals exhibit symptoms similar to those of upper respiratory tract infections, including fever, fatigue, cough, and myalgia.^[2]^ While the majority recover within a few weeks, a subset of individuals experiences long-lasting symptoms and complications, a condition now termed long-COVID that affects a significant proportion of COVID-19 survivors.^[3]^ Beyond its immediate health implications, COVID-19 has been implicated in altering the immune landscape of affected individuals, potentially influencing the risk and progression of cancers.^[4]^ Evaluating these long-term effects is essential for designing effective public health interventions and promoting widespread health betterment.

Cancer, as the second leading cause of death globally, poses a significant public health challenge, particularly for patients whose compromised immune systems increase their vulnerability to severe outcomes during the COVID-19 pandemic.^[5]^ SARS-CoV-2 infection can adversely impact their immune status, disease progression, and routine treatment.^[6]^ Breast cancer is the most frequent cancer diagnosis and the main cause of cancer mortality in women globally, with the HER2-positive subtype reported in 15% to 20% of primary breast cancer.^[7]^ Several studies have shown a close relationship between HER2-positive breast cancer and COVID-19 susceptibility and severity.^[8–10]^ The biological plausibility of this association may be rooted in the immune dysregulation caused by COVID-19, which can alter the tumor immune microenvironment, potentially affecting tumor growth and response to therapy. Additionally, inflammatory cytokines released during severe COVID-19 infection might interact with HER2 signaling pathways, thereby influencing cancer progression and patient prognosis.^[11]^ Immune dysregulation caused by COVID-19 may have a consequential impact on breast cancer, including potential alterations in tumor immune microenvironment and patient prognosis.^[12]^ However, traditional observational studies are biased by unmeasured confounding factors, making it difficult to speculate on the causal relationship between COVID-19 infection and the risk of HER2-positive breast cancer.^[13]^

Mendelian randomization (MR) is an innovative approach that leverages genetic variants to investigate causal relationships between complex traits, thereby reducing biases such as confounding and reverse causation often seen in observational studies. In this study, we utilize MR to explore the potential causal link between genetically predicted susceptibility to COVID-19 and the risk of developing HER2-positive breast cancer. By integrating genetic data and advanced statistical techniques, MR provides a robust framework to assess these associations more rigorously. Our findings aim to shed light on the intricate interplay between COVID-19 susceptibility and the risk of HER2-positive breast cancer, offering valuable insights that could inform clinical practice, public health policies, and future research endeavors.

## 2. Materials and methods

### 2.1. Study design

**Figure 1.**
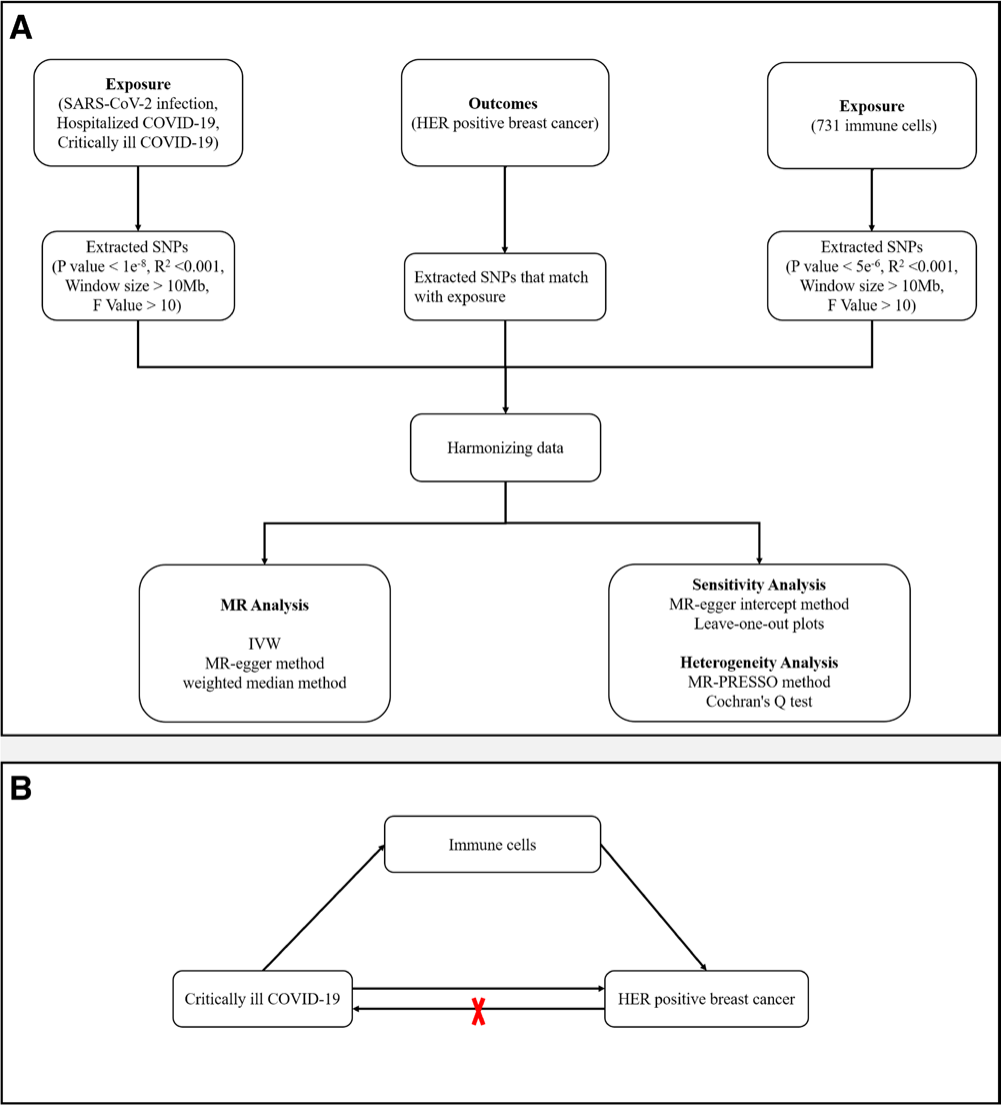
Flowchart describing the research process.

This study is designed according to the STROBE-MR guidelines^[14]^ to make sure that we have done every step of MR analysis. Our complete study design is shown in Figure 1. Two-sample MR study was designed to explore the causal effects and immune cells between COVID-19 and the risk of HER2-positive breast cancer. Our analysis began with an assessment of the causal impact of SARS-CoV-2 infection, hospitalized COVID-19, as well as critically ill COVID-19 on HER2-positive breast cancer, followed by an investigation into the immune cells significantly impacting HER2-positive breast cancer incidence. Next, the single nucleotide polymorphisms (SNPs) related to immune cells were selected as intermediaries. Finally, through mediation analysis, we explored the mediate effect of immune cells between COVID-19 and HER2-positive breast cancer risk.

### 2.2. Data sources and selection of instrumental variables (IVs)

The genome-wide association study (GWAS) data of SARS-CoV-2 infection (N = 2,597,856), hospitalized COVID-19 (N = 2,095,324), and critically ill COVID-19 (N = 1,086,211) were obtained from the COVID-19 Host Genetics Initiative GWAS meta-analysis, round 7 (https://www.covid19hg.org/results/r7/). SNPs that have strong correlations to COVID-19 (*P*-value < 5 × 10^-8^) were retained as IVs. The clump was performed to rule out linkage disequilibrium with *r*^2^ < 0.001 and physical distance within 10,000 kb to ensure the independence of IVs^[15,16]^ (Table S1, Supplemental Digital Content, https://links.lww.com/MD/O854). The summary GWAS statistics of 731 immune cells (N = 3757) were obtained from the MRCEU open database (accession numbers ranging from GCST90001391–GCST90002121).^[17]^ SNPs were selected based on their correlation with the immune cells using the following criteria: *P*-value < 5 × 10^-6^, distance greater than or equal to 10,000 kb, and exclusion of SNPs with linkage disequilibrium (*r*^2^ < 0.001) (Tables S2 and S3, Supplemental Digital Content, https://links.lww.com/MD/O854). The GWAS summary results for HER2-positive breast cancer (N = 103,530) were obtained from the Medical Research Council Integrative Epidemiology Unit OpenGWAS project (https://gwas.mrcieu.ac.uk/).^[18]^ HER2-positive breast cancer, cases were defined using standardized clinical criteria across all datasets.^[18]^ To maintain genetic homogeneity, we restricted our analysis to individuals of European ancestry, reducing population-specific genetic variability.

### 2.3. Sensitivity analysis

A series of sensitivity analyses were conducted to evaluate the robustness and validity of the statistically significant causal associations identified in our study. Cochran *Q* test was performed to assess heterogeneity among the selected genetic instruments, where a significant *P*-value indicates the presence of heterogeneity that may suggest pleiotropy. To minimize potential horizontal pleiotropic effects and ensure the specificity of our genetic instruments, we employed several sensitivity analyses, including the weighted median, MR-Egger regression, and MR Pleiotropy Residual Sum and Outlier (MR-PRESSO). These methods help identify and adjust for pleiotropic SNPs that could bias the results. Additionally, a leave-one-out analysis was conducted to determine whether any single SNP disproportionately influenced the causal estimates by systematically removing each SNP 1 at a time. Collectively, these sensitivity analyses reinforce the reliability of our findings by confirming that the observed associations are not driven by heterogeneity or pleiotropic effects among the genetic instruments.

### 2.4. Statistical analysis

MR analysis was conducted to assess the causal relationship between COVID-19 and the risk of HER2-positive breast cancer (Fig. 1A) and explored potential mediation effect (Fig. 1B). Causal associations were estimated through IVW, MR-Egger, and weighted median method. MR results were expressed as odds ratios (OR) with 95% confidence intervals (CI). *P*-value < .05 was considered statistically significant. All analyses were performed using TwoSampleMR (version 0.5.6) in R (version 4.2.3) packages.

## 3. Results

### 3.1. Causal effects of COVID-19 on HER2-positive breast cancer

**Figure 2.**
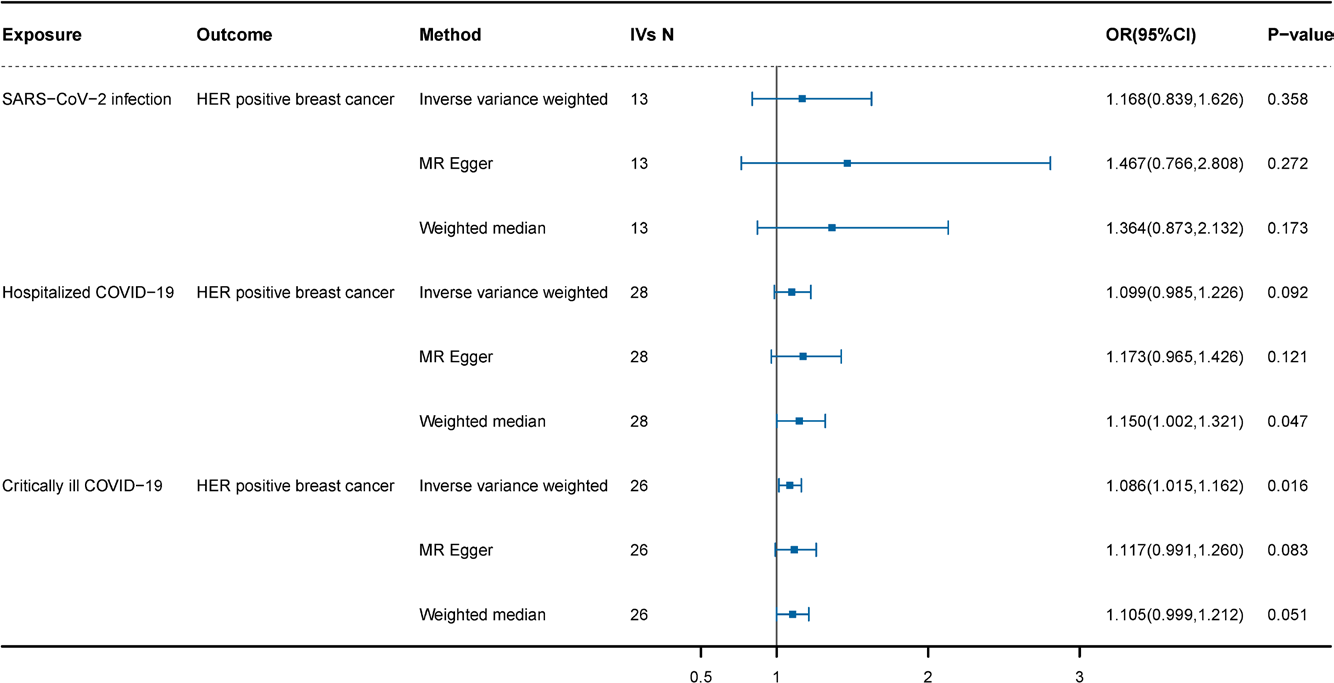
Analysis of the causal effects of COVID-19 on HER2-positive breast cancer. COVID-19 = coronavirus disease 2019.

Following established quality control criteria, 13, 28, and 26 SNPs associated with SARS-CoV-2 infection, hospitalized COVID-19, and critically ill COVID-19 were selected as IVs respectively (Table S1, Supplemental Digital Content, https://links.lww.com/MD/O854). The results from the IVW showed genetic liabilities to critically ill COVID-19 were significantly causally associated with the increased risk of HER2-positive breast cancer (OR = 1.086, 95% CI = 1.015–1.162, *P* = .016), There were no causal associations between hospitalized COVID-19 (OR = 1.099, 95% CI = 0.985–1.226, *P* = .092) and HER2-positive breast cancer. Similarly, no causal associations between SARS-CoV-2 infection (OR = 1.168, 95% CI = 0.839–1.626, *P* = .358) and HER2-positive breast cancer were observed. The results from the MR-Egger, weighted median estimations demonstrated a similar trend, although there was no statistical significance (Fig. 2).

To further evaluate the precision of the results, a series of sensitivity analyses were conducted, including the Cochran *Q* test, MR-Egger intercept analysis, and MR-PRESSO global test. The Cochran *Q* test indicated no evidence of heterogeneity. Similarly, the MR-Egger intercept analysis did not detect horizontal pleiotropy, consistent with findings from the MR-PRESSO global test, enabling us to draw robust conclusions (Table S4, Supplemental Digital Content, https://links.lww.com/MD/O854). Additionally, the leave-one-out method was applied to sequentially exclude SNPs to determine if the causal association was driven by any single IV. The results of these analyses, including funnel plots, forest plots, scatter plots, and leave-one-out tests are presented in the supplement (Figures S1–S12, Supplemental Digital Content, https://links.lww.com/MD/O855).

### 3.2. Causal effects of critically ill COVID-19 on 731 immune cells

**Figure 3.**
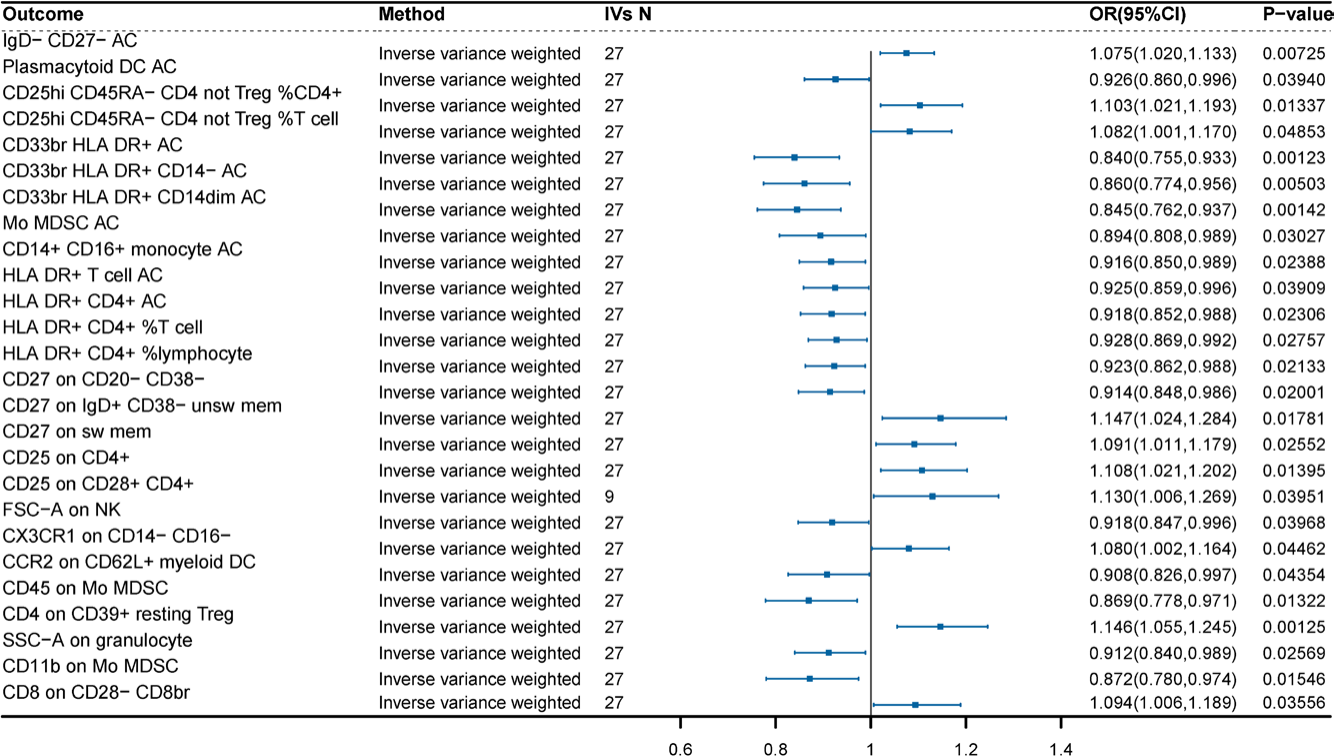
Analysis of the causal effects of COVID-19 on immune cells by IVW method. COVID-19 = coronavirus disease 2019.

To explore the causal effects of critically ill COVID-19 on 731 immune cells, MR analysis was performed (Fig. 3). The IVW method indicated that genetic liabilities to critically ill COVID-19 were significantly positively associated with 9 immune cells (OR = 1.080–1.126, *P* < .05), including CX3CR1 on CD14- CD16-, CD25hi CD45RA- CD4 not Treg %T cell, CD27 on sw mem, CD8 on CD28- CD8br, CD25hi CD45RA- CD4 not Treg %CD4^+^, CD25 on CD4^+^, CD25 on CD28^+^ CD4^+^, CD4 on CD39^+^ resting Treg, as well as CD27 on IgD + CD38- unsw mem (memory B cells). Additionally, genetic liabilities to critically ill COVID-19 were significantly negatively associated with 17 immune cells (OR = 0.839–0.928, *P* < .05), including CD33br HLA DR^+^ AC, CD33br HLA DR^+^, CD14dim AC, CD33br HLA DR^+^ CD14- AC, CD45 on Mo MDSC, CD11b on Mo MDSC, Mo MDSC AC, CCR2 on CD62L^+^ myeloid DC, SSC-A on granulocyte, CD27 on CD20- CD38-, CD14^+^ CD16^+^ monocyte AC, HLA DR^+^ CD4^+^ AC, FSC-A on NK.

HLA DR^+^ CD4^+^ %lymphocyte, IgD- CD27- AC (atypical memory B cells), HLA DR^+^ T cell AC, Plasmacytoid DC AC, and HLA DR + CD4^+^ %T cell. In the sensitivity analyses, the P-values for the pleiotropy tests (MR-Egger intercept test and MR-PRESSO global test) and the heterogeneity assessment (Cochran *Q*-statistic) were all above 0.05, demonstrating the robustness of the results (Table S5, Supplemental Digital Content, https://links.lww.com/MD/O854).

### 3.3. Identification of immune cells with mediating effects

**Figure 4.**
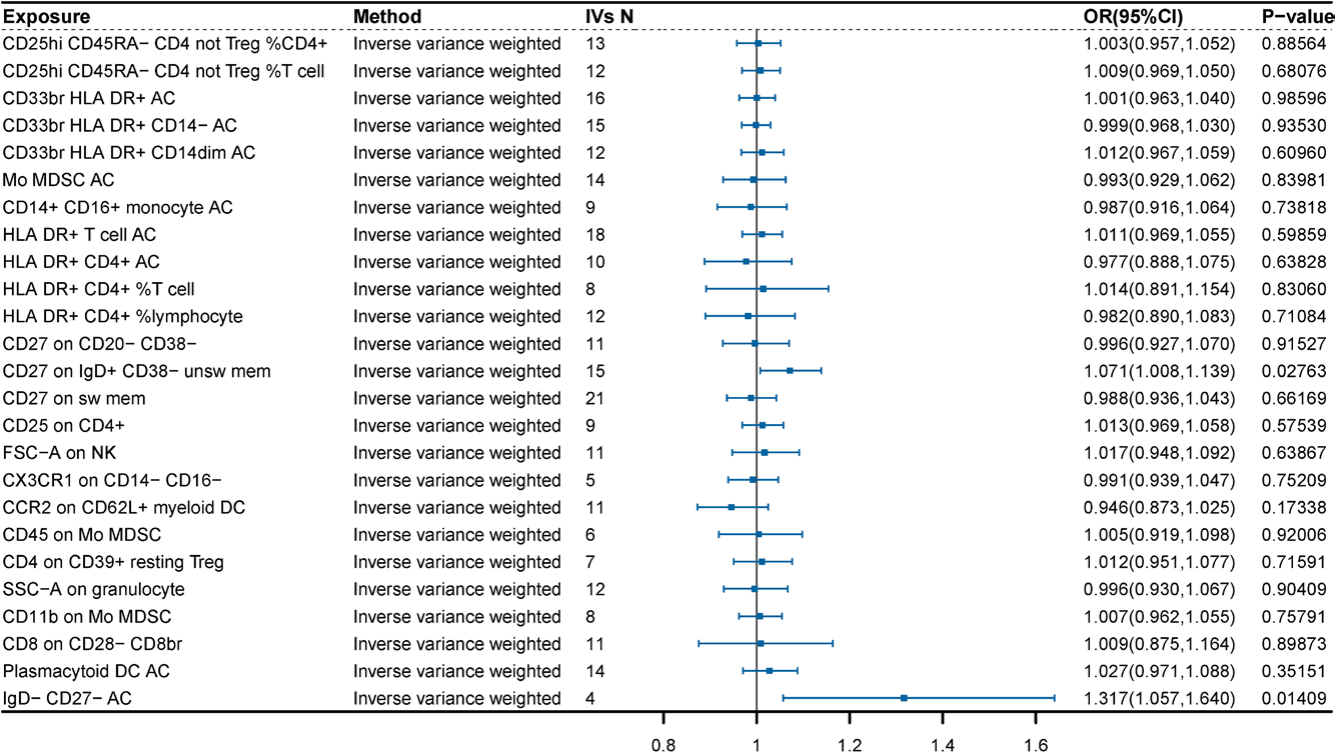
Analysis of the causal effects of immune cells on HER2-positive breast cancer risk by IVW method.

**Figure 5.**
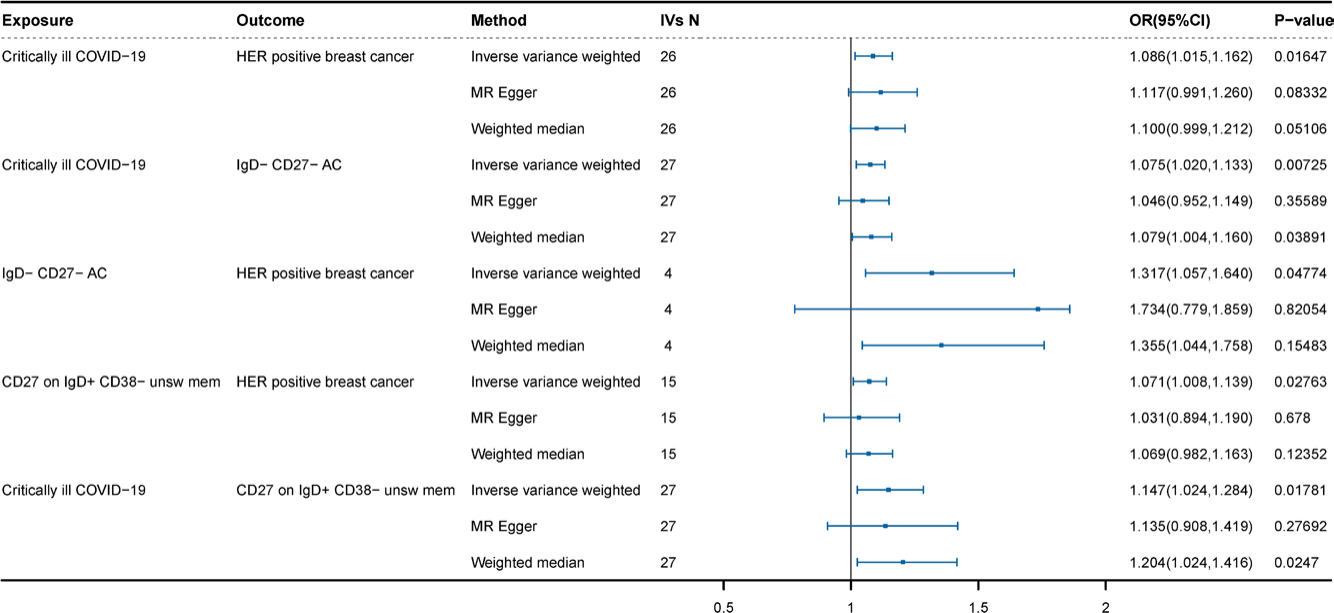
The mediation effect of critically ill COVID-19 risk on HER2-positive breast cancer risk with immune cells. COVID-19 = coronavirus disease 2019.

Following no reverse causation between critically ill COVID-19 and the risk of HER2-positive breast cancer (OR = 1.001, 95% CI = 0.948–1.054, *P* = .994), we sought to identify immune cells with the mediating effect of critically ill COVID-19 on the risk of HER2-positive breast cancer. Initially, we performed MR analysis to screen the causal effects of 26 identified immune cells on the risk of HER2-positive breast cancer (Fig. 4). Ultimately, the analysis results from IVW showed that the genetic prediction of IgD- CD27- AC (atypical memory B cells) (OR = 1.317; 95% CI = 1.057–1.640; *P* = .014) and CD27 on IgD + CD38- unsw mem (OR = 1.071; 95% CI = 1.008–1.139; *P* = .027) were associated with a decreased risk of HER2-positive breast cancer. The sensitivity analysis revealed no significant heterogeneity or horizontal pleiotropy (*P* > .05) (Table S6, Supplemental Digital Content, https://links.lww.com/MD/O854). Therefore, a positive causal relationship was identified between critically ill COVID-19 and IgD- CD27- AC (atypical memory B cells), with the latter also exhibiting a positive causal relationship with the risk of HER2-positive breast cancer (*P* < .05). Moreover, critically ill COVID-19 exhibited causal relationships with CD27 on IgD + CD38- unsw mem, and the latter also demonstrated a positive causal relationship with the risk of HER2-positive breast cancer. These findings suggest that genetic liabilities to critically ill COVID-19 increased the risk of HER2-positive breast cancer by activating IgD- CD27- AC and CD27 on IgD + CD38- unsw mem (Fig. 5).

## 4. Discussion

The COVID-19 pandemic has posed unprecedented public health challenges, characterized by a novel virus with limited epidemiological data and uncertain management protocols. Current observational studies revealed a decline in the number of new cancer diagnoses during the pandemic, with a shift towards more advanced stages at the time of diagnosis compared to the pre-pandemic period.^[19]^ While COVID-19-induced inflammation may affect tumor cells and their microenvironment, the specific effects of COVID-19 on breast cancer remain unknown. Therefore, we employed the MR method to explore potential causal relationships between COVID-19 and HER2-positive breast cancer. This methodological approach aims to provide insights that can guide health promotion strategies during the ongoing and future stages of the pandemic.

We found that critically ill COVID-19 had a causal association with an increased risk of HER2-positive breast cancer, indicating individuals genetically predisposed to severe manifestations of COVID-19 potentially carry a higher risk of developing HER2-positive breast cancer. It may be related to the high expression level of ACE2 in HER2-positive breast cancer, which is positively correlated with EGFR expression and is associated with worse outcomes.^[20]^ Similarly, Zhang et al reported on the clinical characteristics and outcomes of 35 breast cancer patients and found that cancer patients experienced more severe COVID-19 and had a worse prognosis compared to non-cancer patients infected with the virus.^[21]^ Notably, this association is absent in individuals with mere SARS-CoV-2 infection or those hospitalized due to COVID-19 but not critically ill, underscoring the specificity and severity of the interaction. Moreover, 9 immune cells significantly positively associated with genetic liability to critically ill COVID-19 paves the way for understanding the immune-mediated pathways underpinning this relationship. Particularly, the identification of IgD- CD27- AC and CD27 on IgD + CD38- unsw mem as mediators signals towards intricate immune responses catalyzed by severe COVID-19 that might augment oncogenic processes in HER2-positive breast cancer.

Critically ill patients with COVID-19 usually experience a state called “cytokine release syndrome,” characterized by an excessive release of pro-inflammatory cytokines like IL-6, TNF-α, and IL-1β, thus recruiting monocytes, neutrophils and T cells to the lungs.^[22]^ This hyperinflammatory state can create a tumor-promoting environment and exacerbate tumor growth and metastasis.^[23]^ The elevated levels of pro-inflammatory cytokines can promote oncogenic signaling pathways, enhance tumor cell proliferation, and increase resistance to apoptosis.^[24]^ Critically ill COVID-19 patients experience more profound immune dysregulation, including heightened cytokine storms, extensive lymphopenia, and severe T-cell exhaustion.^[22,25]^ These extreme immune alterations contribute to a tumor-promoting microenvironment, facilitating HER2-positive breast cancer progression. Additionally, the genetic variants that predispose individuals to severe COVID-19 may overlap more significantly with those involved in HER2-related oncogenic pathways, thereby strengthening the causal link. These extreme immune alterations can create a tumor-promoting microenvironment that is not as pronounced in milder or general COVID-19 cases. Moreover, such an environment promotes angiogenesis, facilitating the formation of new blood vessels that supply the growing tumor with essential nutrients and oxygen, thereby supporting tumor expansion and enhancing its metastatic potential.^[26]^ In addition, the oxidative stress and DNA damage associated with chronic inflammation can lead to genetic mutations, further driving HER2-positive breast cancer progression and metastasis.^[27]^

Furthermore, severe COVID-19 is associated with immune dysregulation, including lymphopenia and functional exhaustion of T cells and NK cells, and a dysregulated activation of monocytes, neutrophils, and tissue macrophages.^[28,29]^ Disruptions in these immune cell populations could impair the immune system’s capacity to mount effective anti-tumor responses, thereby allowing for unchecked cancer progression.^[30]^ Cancer cells frequently exploit immune checkpoint pathways to evade detection, and severe COVID-19 may alter the regulation of these pathways, thereby exacerbating the tumor’s immune evasion.^[31]^ This alteration can be either direct, due to viral interactions, or indirect, via prolonged systemic inflammation leading to immune exhaustion.^[32]^ Moreover, the interplay between SARS-CoV-2 and cellular signaling pathways involved in HER2-positive breast cancer could further enhance oncogenic processes, potentially leading to more aggressive tumor phenotypes. Our study identified 2 immune cell subsets, specifically IgD- CD27- AC and CD27 on IgD + CD38- unsw mem cells, as mediators linking critically ill COVID-19 to HER2-positive breast cancer, indicating that dysregulation of these immune cell subsets may be a possible mechanism through which critically ill COVID-19 influences HER2-positive breast cancer. In addition, critically ill COVID-19 is associated with a variety of lung pathologies, including inflammation-led diffuse alveolar damage and acute respiratory distress syndrome.^[33]^ In critically ill COVID-19 patients, hypoxic microenvironments may induce cellular dormancy or promote the emergence of an aggressive, drug-resistant phenotype, thereby increasing the potential for subsequent tumor relapse.^[34]^ Pathways involving CD27 and IgD- CD27- AC cells are of particular interest; however, current literature lacks studies directly examining their roles in HER2-positive breast cancer

A common limitation in MR studies is horizontal pleiotropy, which is difficult to resolve completely, even with sensitivity analyses that use various assumptions, because it’s so widespread throughout the genome. The absence of sex-stratified data limited our ability to investigate potential differences between men and women. Our study focused on European populations, and the generalizability of our findings to other populations may be limited. Further research with larger sample sizes and a broader range of ethnic backgrounds is needed to confirm these results.

## 5. Conclusion

The present study suggested that the genetic liability to critically ill COVID-19 increased the risk of HER2-positive breast cancer possibly via immune cells, providing a new pathway for researchers to investigate the biological mechanisms of HER2-positive breast cancer, potentially leading to new therapeutic targets.

## Acknowledgments

We wish to acknowledge all participants and investigators who contributed to the GWAS data.

## Author contributions

**Conceptualization:** Jianfeng Chen, Bingyan Xin, Xiaochuan Zhu.

**Data curation:** Jianfeng Chen, Bingyan Xin, Wenwen Tan, Lan Zhang.

**Formal analysis:** Jianfeng Chen, Bingyan Xin, Wenwen Tan, Zhilian Chen.

**Investigation:** Jianfeng Chen.

**Methodology:** Jianfeng Chen, Bingyan Xin, Zhilian Chen, Lan Zhang.

**Software:** Lan Zhang.

**Supervision:** Xiaochuan Zhu.

**Writing – original draft:** Jianfeng Chen, Bingyan Xin, Wenwen Tan, Zhilian Chen, Lan Zhang, Xiaochuan Zhu.

**Writing – review & editing:** Jianfeng Chen, Bingyan Xin, Wenwen Tan, Zhilian Chen, Lan Zhang, Xiaochuan Zhu.

## Supplementary Material


