# Supplementary material for: Genetic liability to critically ill COVID-19 increased risk of HER2-positive breast cancer through the immune pathway: A Mendelian randomization study
Source: Medicine (Baltimore). 2025 May 9;104(19):e42372. doi: 10.1097/MD.0000000000042372 (PMC12074039; doi:10.1097/MD.0000000000042372)

Figure S1. Forest plot of the causal effect of SARS-CoV-2 infection on HER2-positive breast cancer.

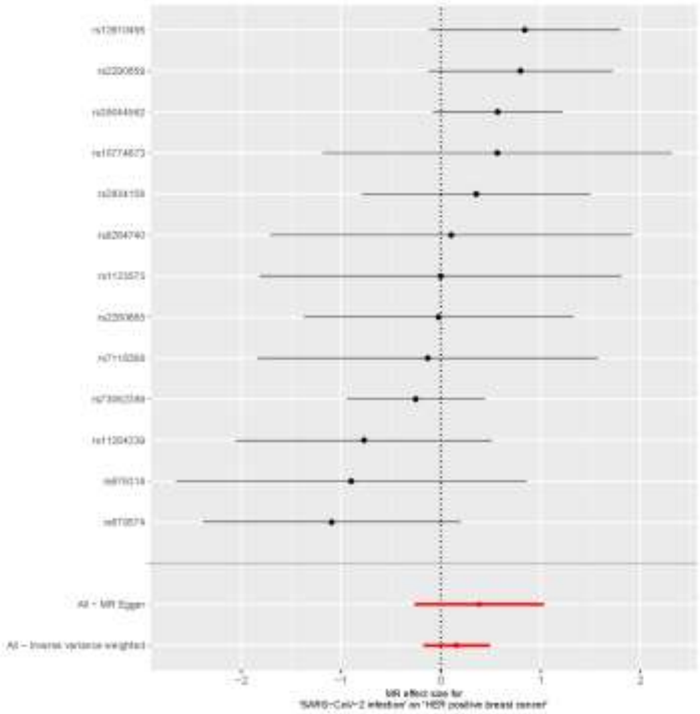

Figure S2. Funnel plot of the causal effect of SARS-CoV-2 infection on HER2-positive breast cancer.

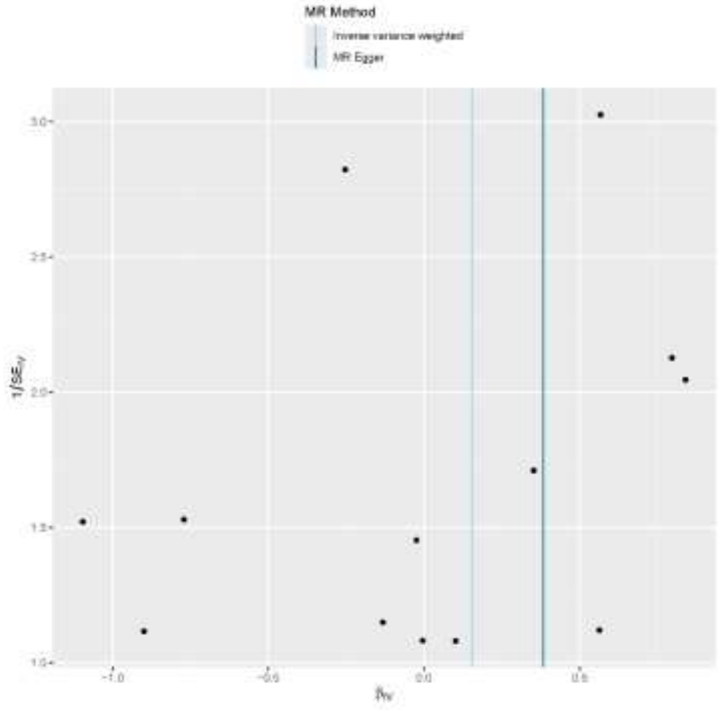

Figure S3. Scatter plots for the causal effect of SARS-CoV-2 infection on HER2-positive breast cancer.

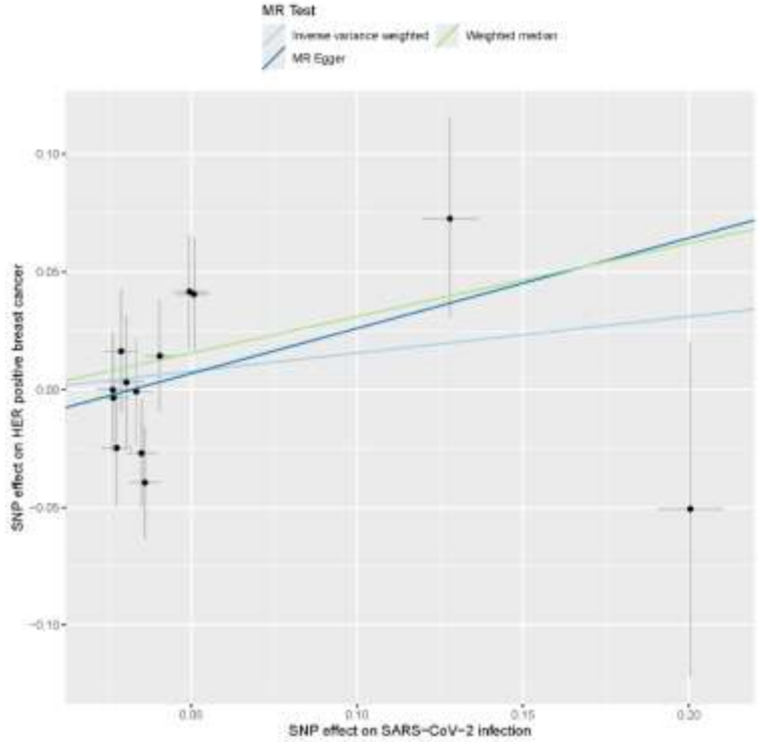

Figure S4. Leave-one-out analysis for the causal effect of SARS-CoV-2 infection on HER2-positive breast cancer.

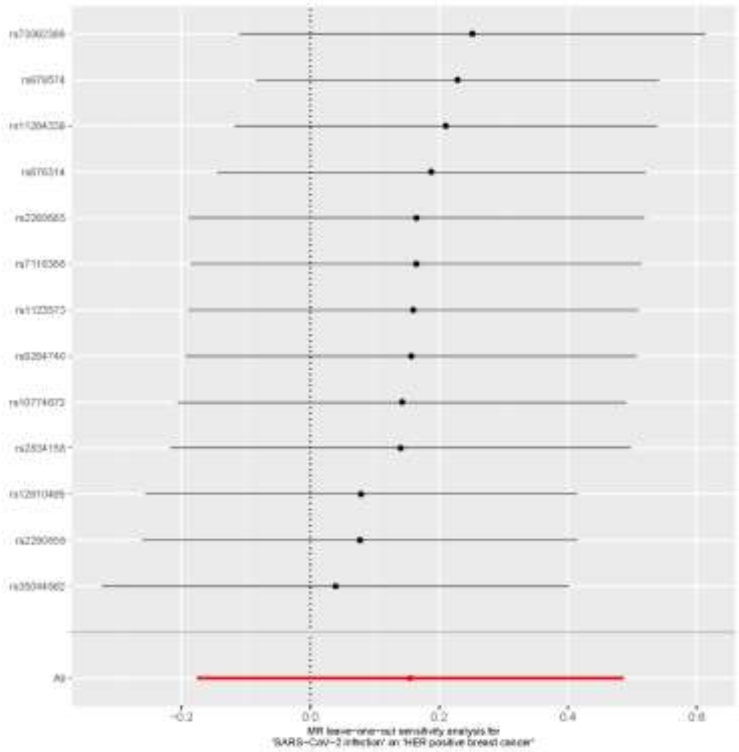

Figure S5. Forest plot of the causal effect of hospitalized COVID-19 on HER2-positive breast cancer.

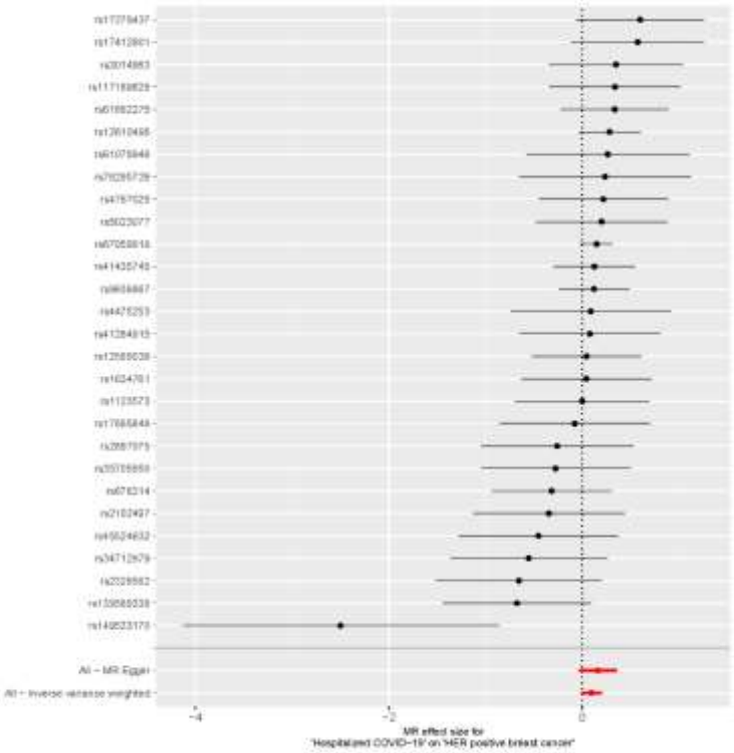

Figure S6. Funnel plot of the causal effect of hospitalized COVID-19 on HER2-positive breast cancer.

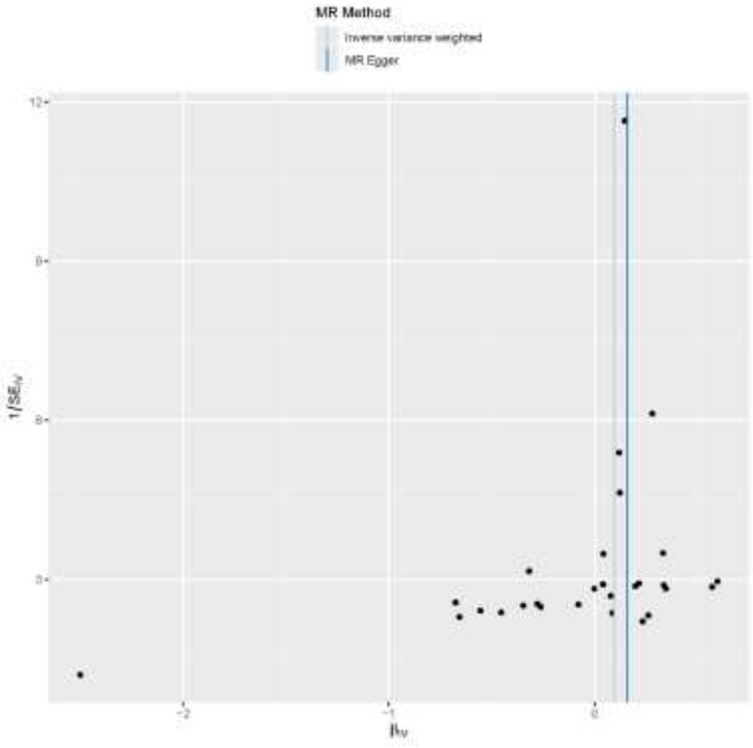

Figure S7. Scatter plots for the causal effect of hospitalized COVID-19 on HER2-positive breast cancer.

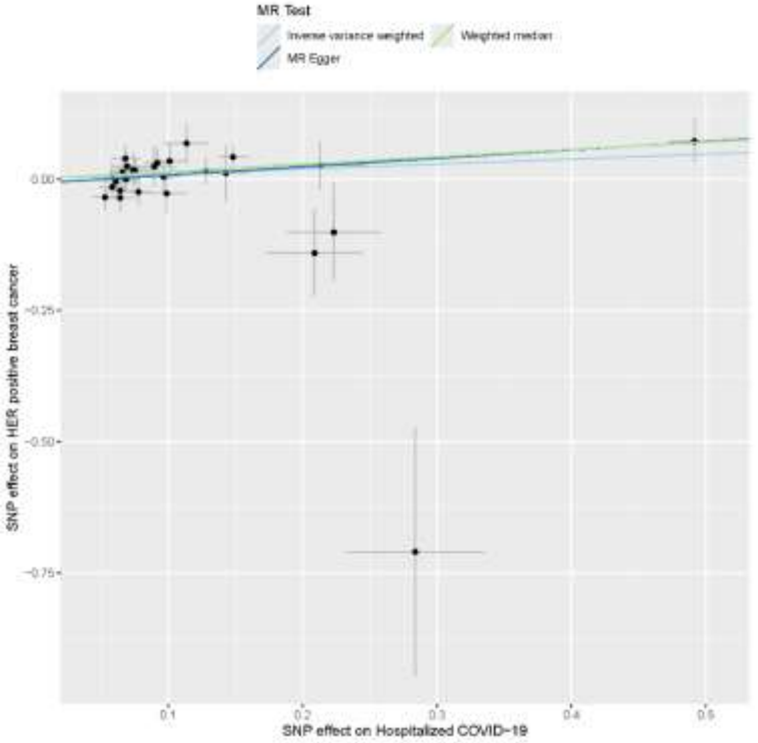

Figure S8. Leave-one-out analysis for the causal effect of hospitalized COVID-19 on HER2-positive breast cancer.

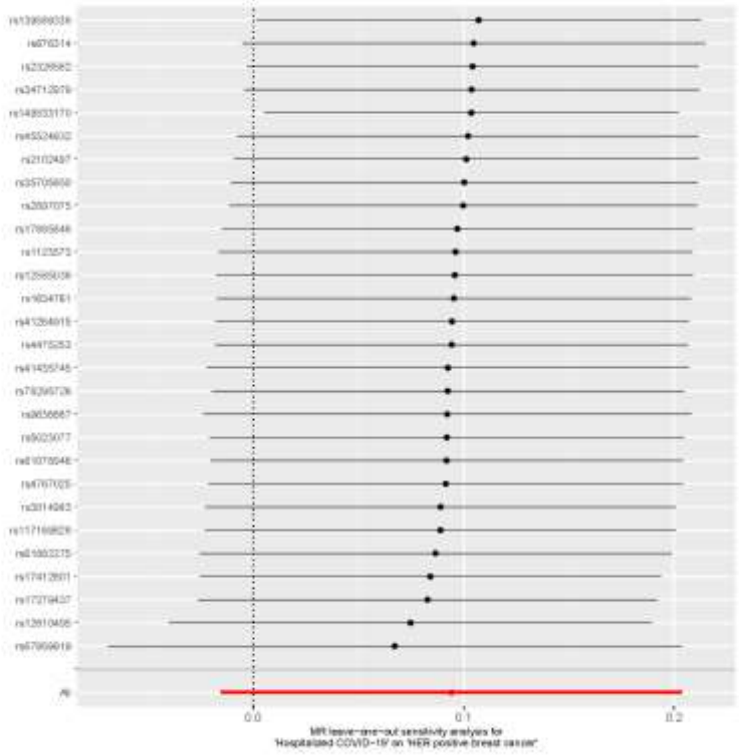

Figure S9. Forest plot of the causal effect of critically ill COVID-19 on HER2-positive breast cancer.

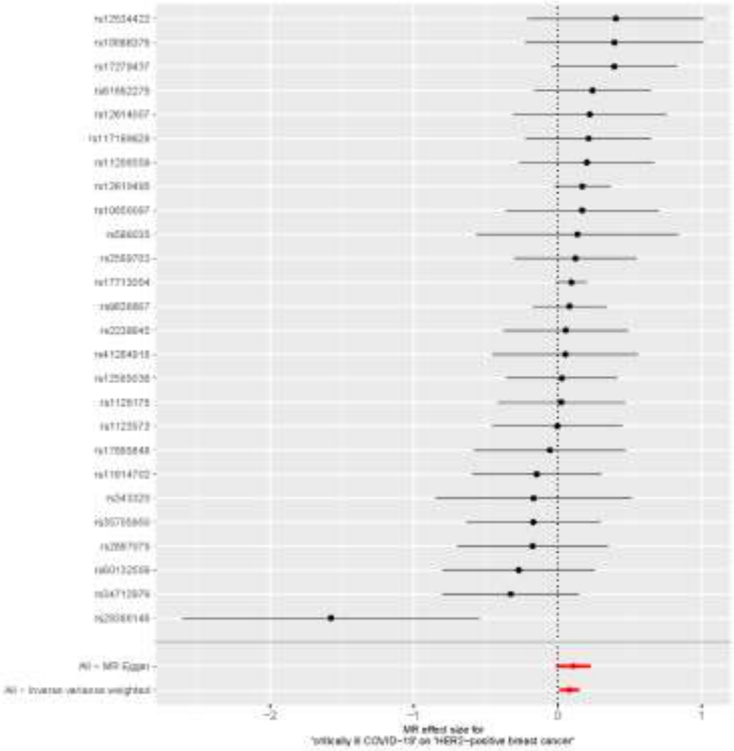

Figure S10. Funnel plot of the causal effect of critically ill COVID-19 on HER2-positive breast cancer.

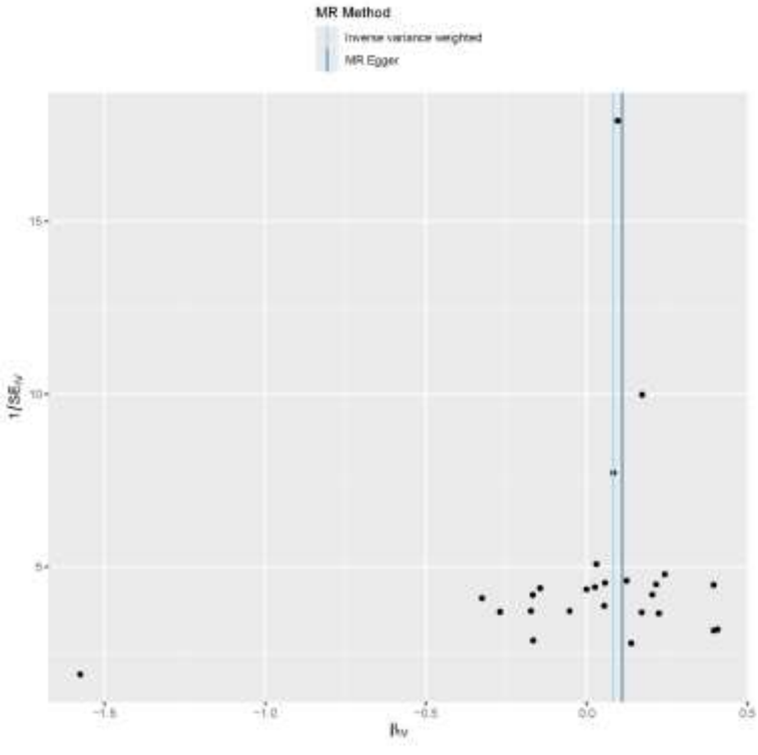

Figure S11. Scatter plots for the causal effect of critically ill COVID-19 on HER2-positive breast cancer.

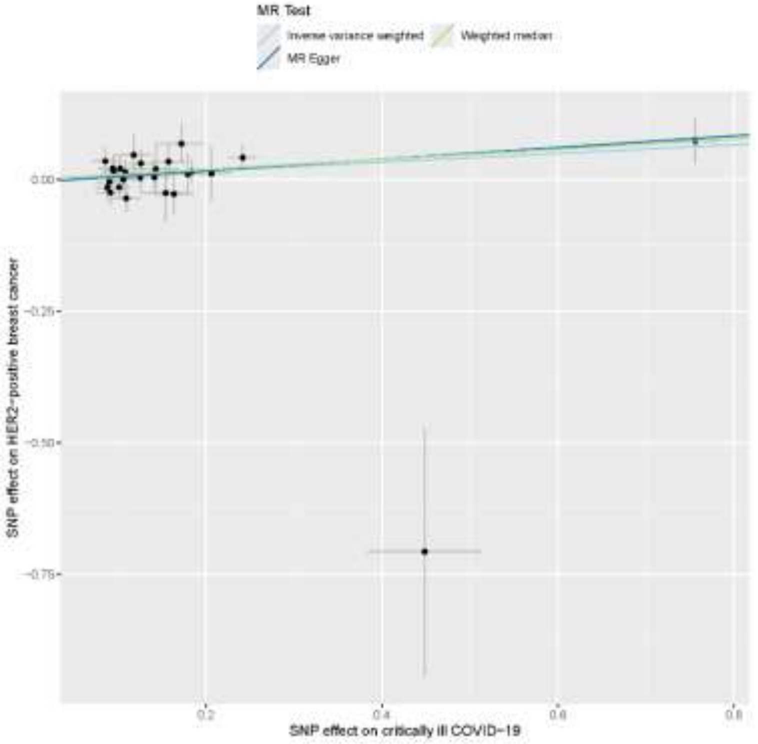

Figure S12. Leave-one-out analysis for the causal effect of critically ill COVID-19 on HER2-positive breast cancer.

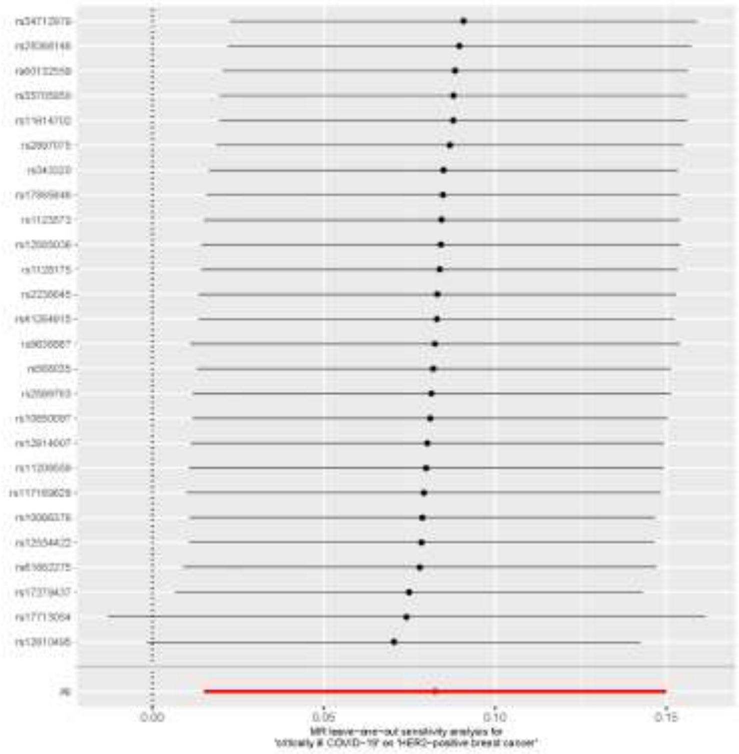

Supplement: Supplementary file 2 [file medi-104-e42372-s002.pdf]
